# Botulinum toxin use in patients with post-stroke spasticity: a nationwide retrospective study from France

**DOI:** 10.3389/fneur.2023.1245228

**Published:** 2023-08-23

**Authors:** Jonathan Levy, Pierre Karam, Anne Forestier, Jean-Yves Loze, Djamel Bensmail

**Affiliations:** ^1^Department of Physical and Rehabilitation Medicine, Raymond-Poincaré Teaching Hospital, AP-HP, Université Paris-Saclay, Garches, France; ^2^Unité INSERM 1179, University of Versailles Saint-Quentin-en-Yvelines, Montigny-Le-Bretonneux, France; ^3^PKCS, Ecully, France; ^4^Ipsen, Boulogne-Billancourt, France

**Keywords:** botulinum toxin, France, spasticity, stroke, care pathway, nationwide data

## Abstract

**Background:**

Current guidelines recommend intramuscular botulinum toxin type A (BoNT-A) injection as first-line treatment for spasticity, a frequent and impairing feature of various central nervous system (CNS) lesions such as stroke. Patients with spasticity commonly require BoNT-A injections once every 3 to 4 months. We conducted a nationwide, population-based, retrospective cohort study, using the French National Hospital Discharge Database (PMSI), to describe BoNT-A use for spasticity in clinical practice in France between 2014 and 2020. The PMSI database covers the whole French population, corresponding to over 66 million persons.

**Methods:**

We first searched the PMSI database for healthcare facility discharge of patients who received BoNT-A injections between 2014 and 2020, corresponding to the first set. For each BoNT-A-treated patient, we identified the medical condition for which BoNT-A may have been indicated. Another search of the PMSI database focused on patients admitted for acute stroke between 2014 and 2016 and their spasticity-related care pathway (second set). Overall, two subpopulations were analysed: 138,481 patients who received BoNT-A injections between 2014 and 2020, and 318,025 patients who survived a stroke event between 2014 and 2016 and were followed up until 2020.

**Results:**

Among the 138,481 BoNT-A-treated patients, 53.5% received only one or two BoNT-A injections. Most of these patients (*N* = 85,900; 62.0%) received BoNT-A because they had CNS lesions. The number of patients with CNS lesions who received ≥1 BoNT-A injection increased by a mean of 7.5% per year from 2014 to 2019, but decreased by 0.2% between 2019 and 2020, corresponding to the COVID-19 outbreak. In stroke survivors (N = 318,025), 10.7% were coded with post-stroke spasticity, 2.3% received ≥1 BoNT-A injection between 2014 and 2020, and only 0.8% received ≥3 injections within the 12 months following BoNT-A treatment initiation, i.e., once every 3 to 4 months.

**Conclusion:**

Our analysis of the exhaustive PMSI database showed a suboptimal implementation of BoNT-A treatment recommendations in France. BoNT-A treatment initiation and re-administration are low, particularly in patients with post-stroke spasticity. Further investigations may help explain this observation, and may target specific actions to improve spasticity-related care pathway.

## 1. Introduction

Spasticity can be defined as a disordered sensori-motor control, resulting from an upper motor neuron lesion, and presenting as an intermittent or sustained involuntary activation of muscles (1). It is a frequent and impairing feature of various central nervous system (CNS) pathologies, in particular stroke (2, 3). If left untreated, spasticity can adversely influence quality of life, since it may interfere with routine task performance, contribute to the development of muscle contractures and pain, and make hygiene and self-care difficult (4). Despite the ease of diagnosis, effective spasticity management is often challenging for the clinician, as it is multimodal and tailored to individual patient concerns (5). Treatment options include physical therapy, bracing, neuromuscular electrical stimulation, surgery, and oral and/or injectable medications including baclofen, benzodiazepines, tizanidine, dantrolene, and chemodenervation agents such as botulinum toxin (BoNT) (6, 7).

Among the pharmacological options, intramuscular botulinum toxin type A (BoNT-A) injection is currently recommended as a first-line focal and reversible treatment for spasticity by many national/international guidelines (8), including guidelines from France (9). BoNT-A acts by blocking acetylcholine release at the neuromuscular junction, and, thereby, reduces muscle hyperactivity and spasticity (10). Patients with spasticity usually require repeated BoNT-A intramuscular injections once every 3 to 4 months (9, 11, 12).

Despite the integral role of BoNT-A in the management of spasticity, there are very limited data on its real-world use in patients with spasticity. To our knowledge, no nationwide study on the extent of BoNT-A use for spasticity has been reported to date. More generally, although several researchers have studied the incidence of post-stroke spasticity (13), fewer have evaluated treatment patterns in this patient population. Accordingly, the primary aim of this real-world, population-based analysis of healthcare data was to determine the extent of BoNT-A use across France between 2014 and 2020, particularly in patients with stroke. By having patient data from 2014–2020, we were also able to examine the evolution of spasticity management during France’s first two national lockdowns in 2020 imposed in response to the COVID-19 pandemic and the pandemic’s impact on patient management.

## 2. Materials and methods

### 2.1. Study design and data source

We carried out a nationwide, population-based, retrospective cohort study between 2014 and 2020, using data extracted from the French National Hospital Discharge Database (*Programme de Médicalisation des Systèmes d’Information*, PMSI). This study was conducted in compliance with all French laws and regulations and the Declaration of Helsinki, and was notified to the French Data Protection Agency (*Commission Nationale de l’Informatique et des Libertés*, CNIL). According to the French law and the CNIL, the study did not require formal ethical approval and persons’ informed consents, as it did not involve direct participation of human individuals and all extracted data were anonymised.

The PMSI database is a comprehensive claims database that includes information on diagnoses and procedures. Diagnoses are coded according to the International Classification of Diseases version 10 (ICD-10), either as main diagnosis, related diagnosis, or significantly associated diagnoses. Medical procedures are coded according to the French Joint Classification of Medical Procedures (*Classification Commune des Actes Médicaux*, CCAM).

PMSI is an exhaustive database that covers the whole French population, corresponding to over 66 million persons. It contains standardized discharge summary reports from all inpatients and outpatients discharged from public hospitals as well as from inpatients discharged from private hospitals in France. Each PMSI record provides the following information: administrative information including length of hospital stay and geographic location of the hospital; demographic data (sex, age); and therapeutic procedures coded during each stay. In addition, for inpatients, there is information on medical diagnoses; comorbidities managed during the stay; and costly drugs and implantable medical devices that were administered. The PMSI database also includes an anonymous unique patient identifier, which allows to follow all hospital admissions and stays for each individual patient, even when the patient has been admitted to several healthcare facilities or hospital departments.

The PMSI database does not contain patient charts and does not provide clinical information such as the dosage or effectiveness of administered drugs, the muscles injected, or the goal of the treatment. It also prevents any causal association from being drawn.

### 2.2. Study population

#### 2.2.1. Patients who received at least one BoNT-A injection (first analysis set)

We first searched the PMSI database for healthcare facility discharge of patients (both adult and paediatric) who received at least one intramuscular BoNT-A injection between 2014 and 2020, using the following CCAM codes: PCLB002 and PCLB003. Both codes refer to a single session of BoNT-A injection(s) within striated muscles. For each patient treated with at least one intramuscular BoNT-A injection, we extracted data on all inpatient diagnoses, and inpatient and outpatient stays and performed medical procedures. The conditions for which BoNT-A may have been indicated were then identified using ICD-10 codes from a primary, related, or significantly associated diagnosis.

**Figure 1:**
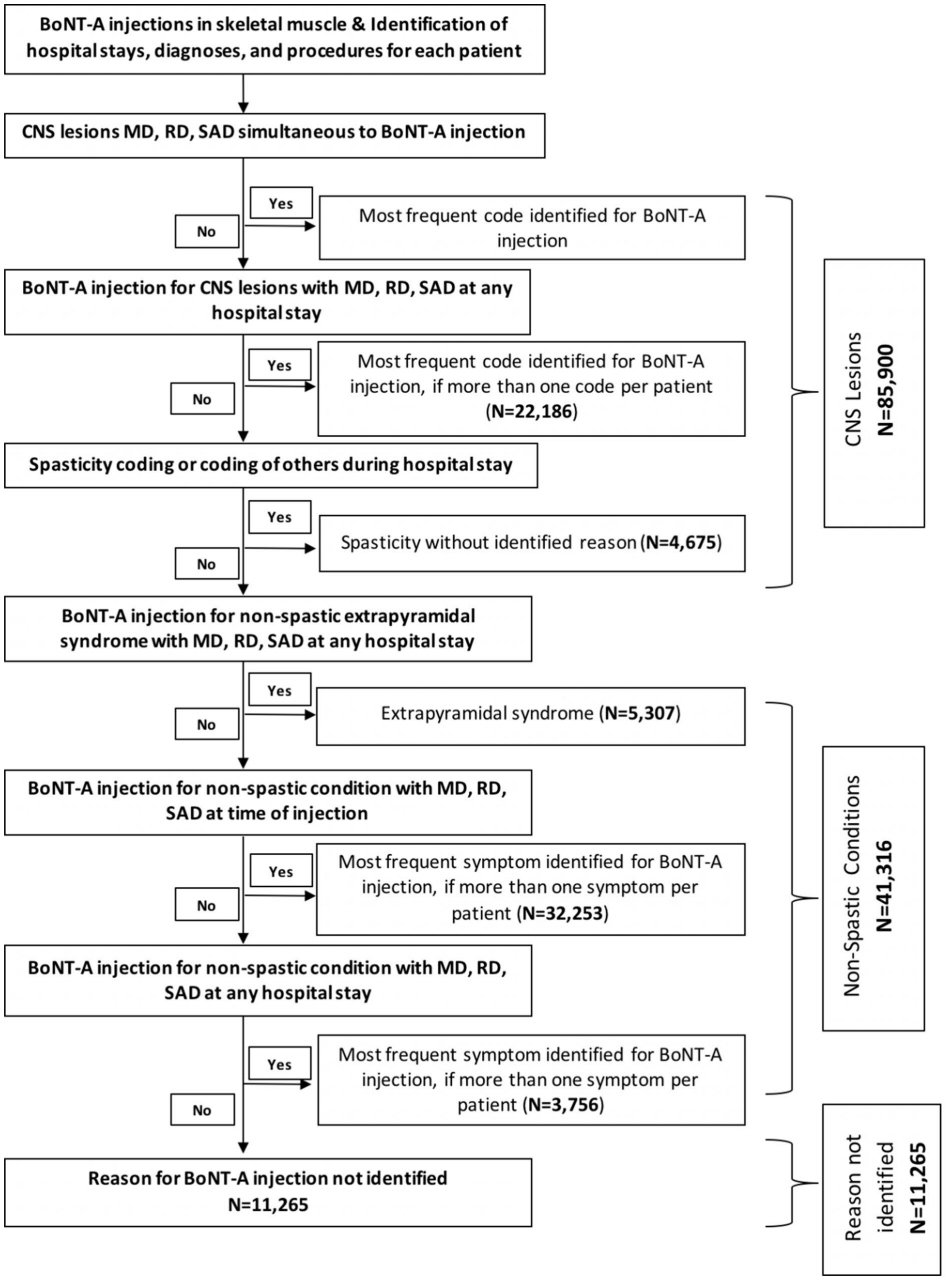
Selection process of patients by reason for botulinum toxin type A (BoNT-A) injection. CNS, central nervous system; MD, main diagnosis; RD, related diagnosis; SAD, significantly associated diagnosis.

Figure 1 illustrates in a flow chart the process of selection of patients by reason for BoNT-A injection. Our selection approach consisted of a step-by-step verification from the most to the least certain diagnosis, in order to minimise data loss and data redundancy/duplication. For instance, from the overall population, i.e., patients who received at least one coded BoNT-A injection, we classified those having simultaneously a coded CNS lesion. Then, we applied another selection criterion to the remaining population to select those who had a CNS lesion coded at any hospital stay between 2014 and 2020 different from the one during which the BoNT-A injection was administered. When BoNT-A was administered in relation to more than one condition in a single patient, only the most frequently coded condition was retained. Hence, each patient had only one condition justifying BoNT-A administration recorded in our analysis.

Patients treated with BoNT-A were also analysed according to their age, sex, and the type of healthcare facility to which they were admitted. Healthcare facilities were classified into the following categories: acute care facilities (*Médecine-Chirurgie-Obstétrique*, MCO); and postoperative and rehabilitation facilities (*Soins de Suite et de Réadaptation*, SSR).

#### 2.2.2. Patients who survived a stroke event between 2014 and 2016 (second analysis set)

The second search of the PMSI database was focused on patients presenting with stroke between 2014 and 2016 to ensure a longitudinal post-stroke care pathway. We identified all patients (both adult and paediatric) who were admitted at least once for acute stroke, and excluded those who died within 6 months of their admission for this event. Patients with stroke who survived after 6 months were followed up until 2020.

### 2.3. Outcomes

In patients who received at least one BoNT-A injection, several outcomes were evaluated, such as the total number of BoNT-A injections between 2014 and 2020 among all patients and among those coded with CNS lesions. The percentage change in the number of BoNT-A injections between 2014 and 2020, and the distribution of BoNT-A injections between acute care facilities and postoperative/rehabilitation facilities were also assessed.

In the specific analysis focused on patients with stroke, we evaluated the prevalence of post-stroke spasticity, the extent of BoNT-A use (at least 1 or 3 injections) in stroke survivors, and the median time from stroke onset to the first BoNT-A injection.

### 2.4. Statistical analysis

Descriptive analyses were performed using number and percentage for categorical variables, and mean, standard deviation, median, and first and third quartiles (Q1–Q3) for continuous variables. All data underlying this publication were provided by the *Agence Technique de l’Information sur l’Hospitalisation* (ATIH). Data analyses were performed by the second author, a biostatistical expert, using the SQL server software. Of note, the expert who carried out the analyses has followed and validated the mandatory training for access to PMSI data. The expert has also made a commitment to the CNIL regarding the confidentiality and independence of the research laboratories and design offices provided for by the decree of July 17, 2017.

## 3. Results

### 3.1. BoNT-A use in patients who received at least one BoNT-A injection

A total of 138,481 patients (both adult and paediatric) who received at least one BoNT-A injection (total of 620,416 injections) were included in the analysis during the study period. Most of these patients (*N* = 85,900; 62.0%) received BoNT-A because they had CNS lesions known to cause spasticity (Table 1). Between 2014 and 2020, more than half of patients (74,123/138,430; 53.5%) received only one (50,654; 36.6%) or two (23,469; 17.0%) BoNT-A injections.

**Table 1:** Types of central nervous system (CNS) lesions for which botulinum toxin type A (BoNT-A) may have been indicated.

CNS lesions known to cause spastic hypertonia and related conditions
Cerebrovascular diseases (including stroke)
Multiple sclerosis (MS)
Congenital CNS disorders (e.g., cerebral palsy, spina bifida)
CNS genetic disorders
Infectious and inflammatory CNS disorders excluding MS
Acquired non-vascular cerebral lesions
Vertebro-medullary lesions
Dementia
Other CNS disorders causing spasticity without well-defined aetiology, excluding anoxia
Spasticity without well-defined aetiology

**Figure 2:**
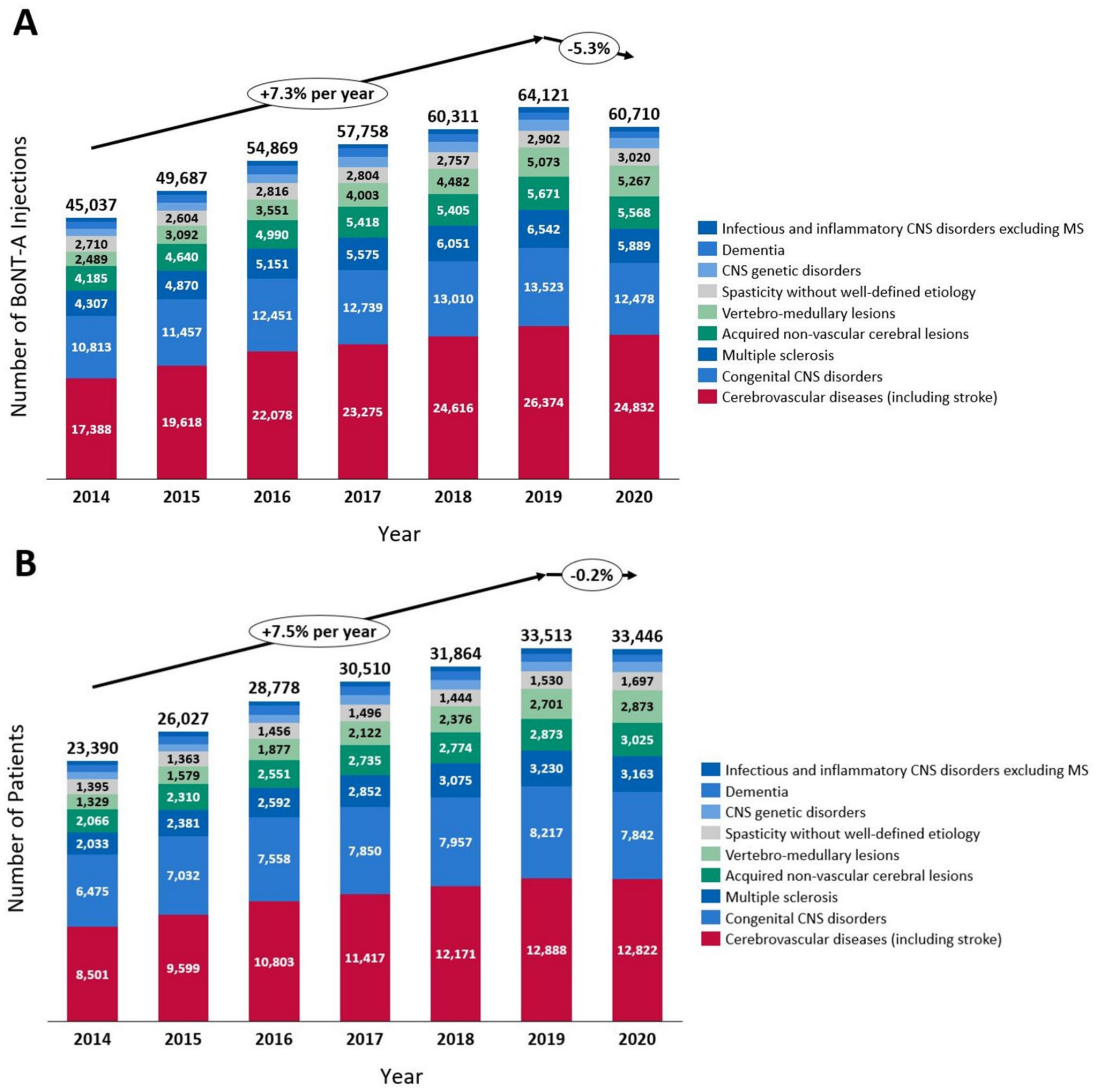
Changes between 2014 and 2020 in the number of botulinum toxin type A (BoNT-A) injections **(A)** in patients with central nervous system (CNS) lesions and in the number of patients **(B)** with CNS lesions treated with BoNT-A. MS, multiple sclerosis.

Overall, the number of BoNT-A injections steadily increased from 69,721 in 2014 to 101,758 in 2019, resulting in a mean increase of 7.9% per year, before plateauing in 2020 compared to 2019, as a small increase of only 1.9% was noted between 2019 and 2020. Specifically, in the 85,900 patients with CNS lesions, the number of BoNT-A injections increased by a mean of 7.3% per year from 2014 to 2019, and decreased by 5.3% from 2019 to 2020 (Figure 2A). The number of patients with CNS lesions who received at least one BoNT-A injection remained however stable during 2019–2020, with a marginal 0.2% decrease (Figure 2B).

**Figure 3:**
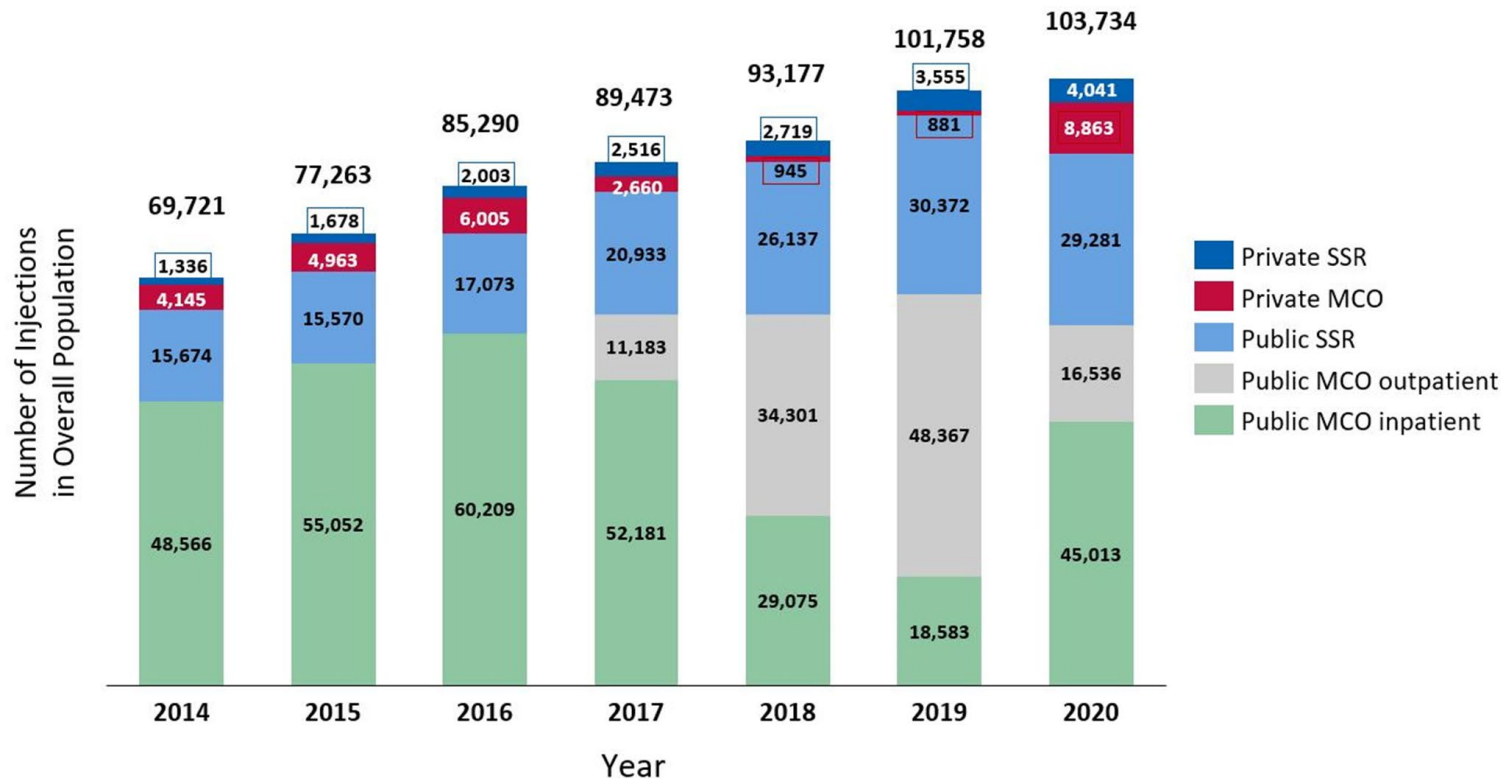
Distribution of the number of botulinum toxin injections in the overall study population, according to healthcare facilities. MCO refers to the acute care facilities, and SSR refers to the postoperative and rehabilitation facilities.

Figure 3 illustrates the distribution of the number of BoNT-A injections per year, according to healthcare facilities. Overall, there was a notably higher number of BoNT-A injections performed in acute care facilities compared to postoperative/rehabilitation facilities. Injections in public acute care facilities, which were exclusively coded in the inpatient setting between 2014 and 2016, dramatically shifted to outpatient care in 2018 and 2019 at the expense of inpatient care, in relation to changes in the invoicing system in France.

### 3.2. BoNT-A use in patients with stroke

**Figure 4:**
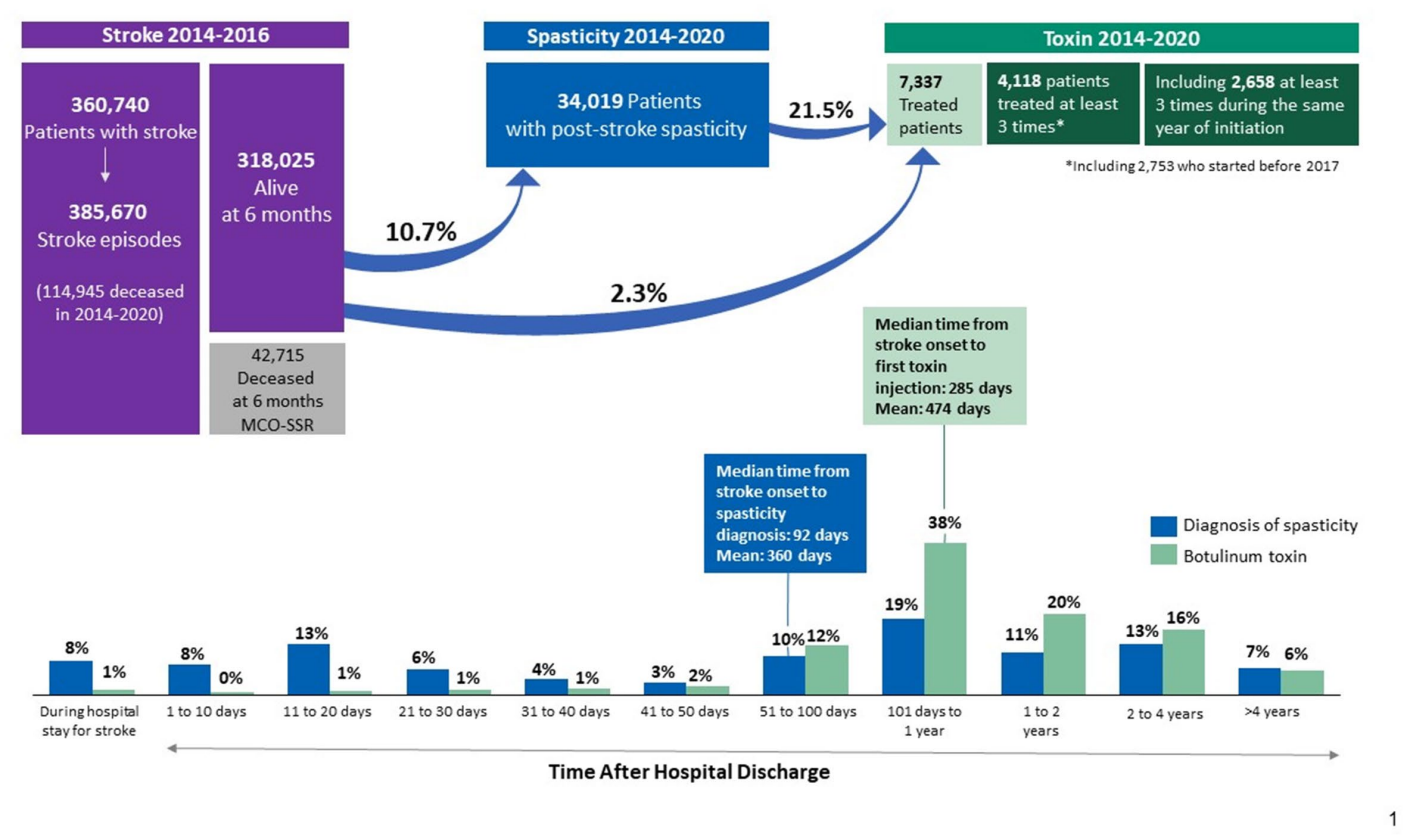
Care pathway of patients with stroke in French hospitals between 2014 and 2020. MCO refers to the acute care facilities, and SSR refers to the postoperative and rehabilitation facilities.

**Figure 5:**
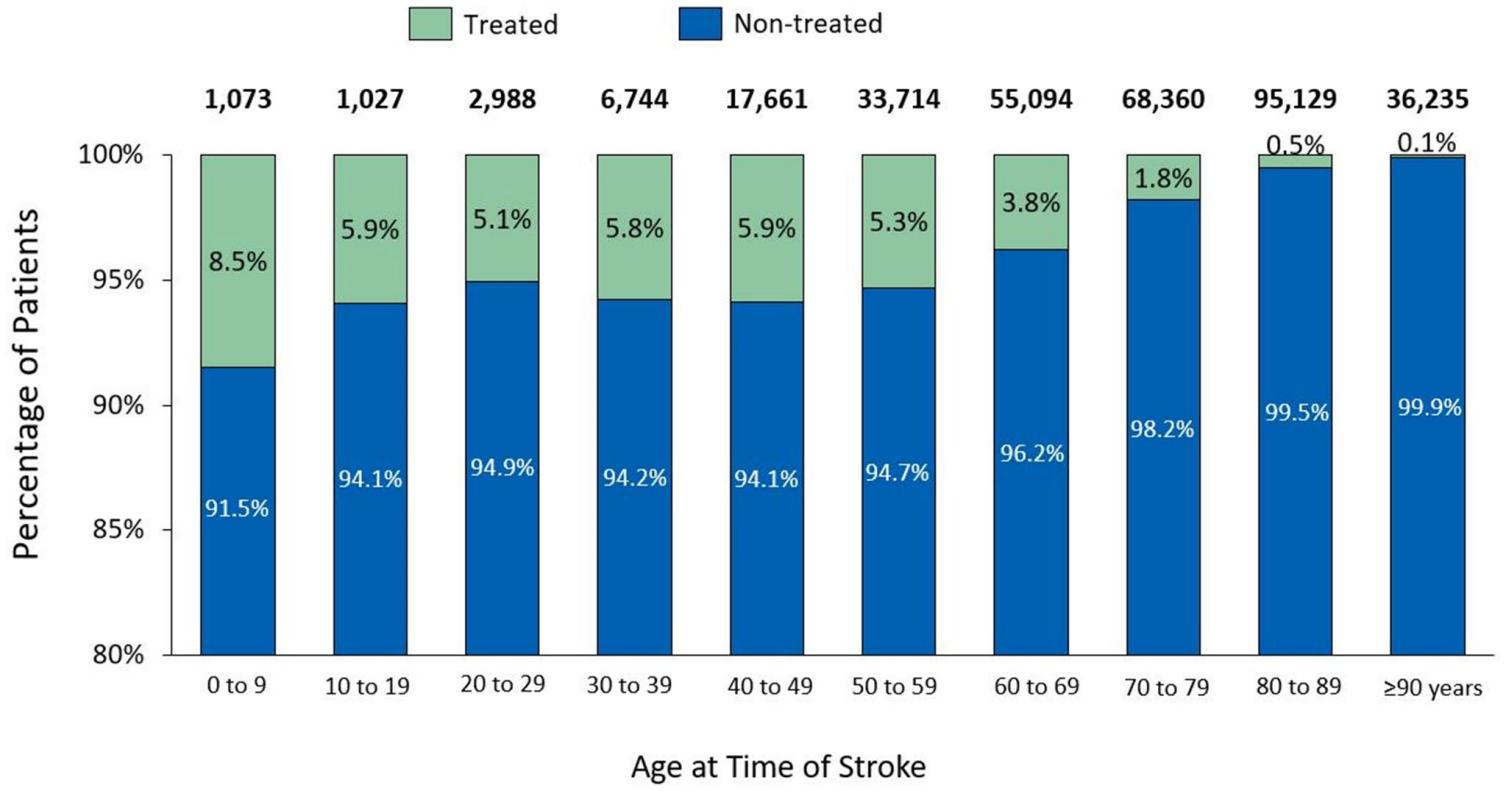
Botulinum toxin treatment rates by age in patients with stroke.

Between 2014 and 2016, a total of 360,740 patients (both adult and paediatric) experienced at least one stroke event (385,670 events in total), 42,715 of whom died within the first 6 months, leaving 318,025 patients to be followed up until 2020. Of these 318,025 stroke survivors, 34,019 (10.7%) were coded with post-stroke spasticity, 7,337 (2.3%) received at least one BoNT-A injection between 2014 and 2020, and 2,658 (0.8%) received at least 3 injections within the 12 months following BoNT-A initiation. The median (Q1–Q3) time from stroke onset to spasticity coding was 92 (17–503) days. Only 21.5% of patients coded with post-stroke spasticity received at least one BoNT-A injection, with the median (Q1–Q3) time between stroke onset and the first BoNT-A injection of 285 (125–670) days (Figure 4). An age-specific analysis indicated a steady decrease in the proportion of stroke patients treated with at least one BoNT-A injection, from 8.5% in patients aged <10 years to 0.1% in those aged >90 years (Figure 5).

**Figure 6:**
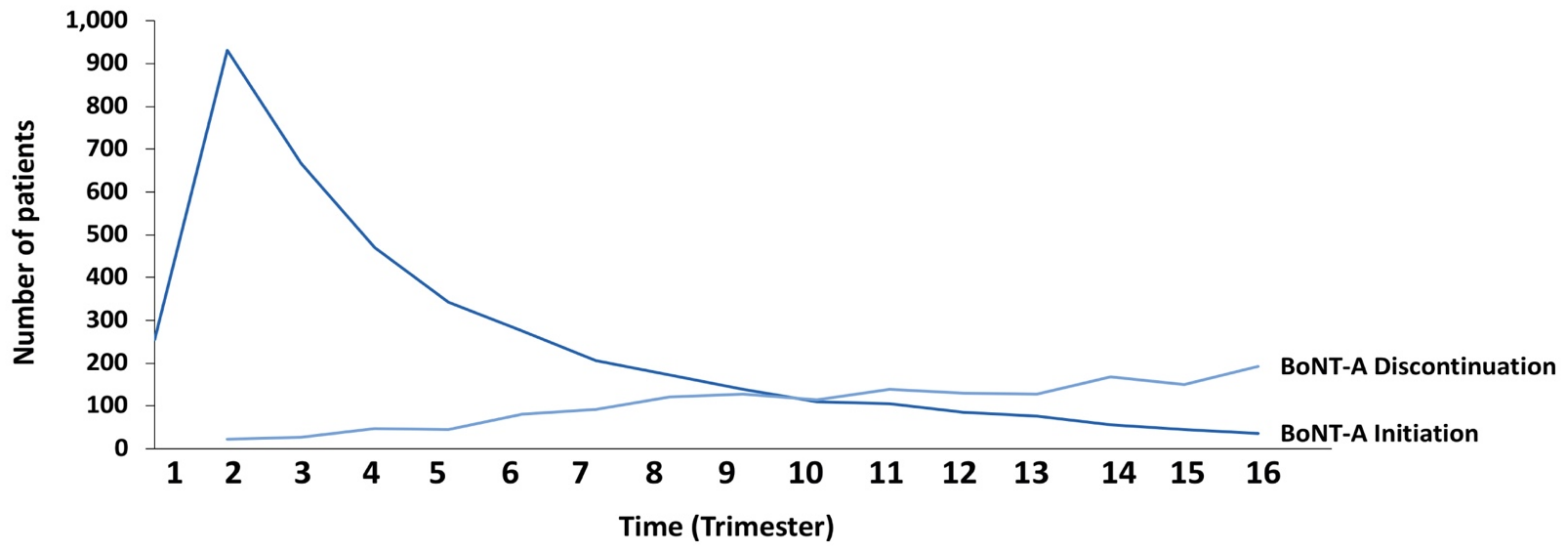
Kinetics of initiation and discontinuation of botulinum toxin type A (BoNT-A) therapy among stroke survivors.

More than half of BoNT-A-treated patients coded with post-stroke spasticity (4,118/7,377; 55.8%) received at least 3 injections between 2014 and 2020. For the 4,118 patients with at least 3 injections, the peak in the number of patients treated with BoNT-A was reached at 9 months following the first injection. Subsequently, the number of patients who discontinued BoNT-A therapy surpassed the number of those who initiated BoNT-A therapy (Figure 6).

## 4. Discussion

BoNT-A, administered as a local intramuscular injection, is considered as a first-line pharmacological treatment of spasticity, with a strong body of evidence for its efficacy and safety in the management of both upper and lower limb spasticity (4, 8, 9, 12, 14–17). Clinical practice guidelines however support the adoption of an individualised, patient-centric approach to the therapeutic use of BoNT-A, as there is no clear evidence to guide the optimal dose of BoNT-A per injection session, injection sites, and the optimal timing and frequency of BoNT-A administration (4, 5, 12, 15, 17). It is recommended to select BoNT-A treatment intervals and duration based on individual patient needs, with keeping in mind that the effect of BoNT-A injections lasts for an average of 3–4 months (12, 15, 17, 18).

Our analysis of the comprehensive French PMSI database aimed to reflect a broad range of BoNT-A treatment practices between 2014 and 2020. To the best of our knowledge, this is the first published study evaluating BoNT-A use on such a large scale, as the PMSI database is one of the world’s largest continuous homogeneous claims databases, covering the whole French population, i.e., over 66 million persons (19). Several informative findings were noted in the present analysis.

Overall, the number of BoNT-A injections notably increased between 2014 and 2019, with a mean increase of 7.3% per year among patients with spasticity due to CNS lesions. However, between 2019 and 2020, we noted a decrease of 5.3% in the number of BoNT-A injections administered. Despite this decrease in the number of injections between 2019 and 2020, the number of patients with CNS lesions who were treated with BoNT-A for spasticity remained stable during this period. Hence, we presume that, during the COVID-19 outbreak in 2020, there were few patients newly admitted to the spasticity clinics in France, and regularly treated patients experienced extended delays in repeated BoNT-A injections. These results are in line with other real-world studies from Italy and Austria conducted in spasticity outpatient clinics, in which the COVID-19 pandemic lockdown had a negative impact on spasticity management, with increased inter-injection intervals among patients treated with BoNT-A for spasticity (20–23).

Among the 318,025 stroke survivors, 10.7% of patients were coded with post-stroke spasticity. This figure concurs with another large analysis based on German statutory health insurance data reporting a spasticity prevalence of 10.2% among survivors within 6 months after stroke (24). In a meta-analysis of 22 cohort studies reporting on the prevalence of post-stroke spasticity, the pooled prevalence of spasticity after stroke was 25.3% and that after the first-ever stroke was 26.7% (13). Overall, the observed prevalence of post-stroke spasticity varies substantially across studies, ranging from 10% up to 92% depending on the sample size and the study methodology (13, 24–26). Furthermore, it has been suggested that estimates of incidence and prevalence of post-stroke spasticity vary widely due to discrepancies in defining and clinically measuring spasticity, as well as variations in severity and chronicity of stroke (27). Another interesting finding of the present study was that the proportion of patients with post-stroke spasticity who received at least one BoNT-A injection was 21.5%. This figure is actually higher than that reported in a national epidemiological study from Sweden by Forsmark et al. (28), in which the mean proportion of patients with disabling spasticity treated with BoNT-A was 9.2%. According to the authors, this low nationwide use of BoNT-A as a treatment of spasticity can be attributed to a lack of consensus on spasticity treatment and insufficient up-to date expertise (28).

Importantly, only 7,337 (2.3%) of the 318,025 stroke survivors in the current study received at least one BoNT-A injection between 2014 and 2020. In addition, only 2,658 (0.8%) received at least 3 injections within the 12 months following BoNT-A initiation, which overall corresponds to a BoNT-A injection given once every 3–4 months, as per guidelines’ recommendations (15). Hence, very few stroke survivors seem to have benefited from BoNT-A therapy, and even fewer received BoNT-A treatment according to recommendations (12, 15, 17). These findings make us speculate about the reasons for this limited use of BoNT-A, particularly since cost is not an issue. In France, all health-care related expenses for chronic diseases such as stroke, including in patients with spasticity, are fully reimbursed by the national health insurance (29). The safety of BoNT-A treatment is also not an issue, since BoNT-A has consistently demonstrated a favourable safety profile in patients with post-stroke spasticity (30, 31). In accordance with the study from Sweden by Forsmark et al. (28), potential reasons behind the reduced use of BoNT-A in the present study may be a limited physicians’ awareness of clinical practice guidelines and of the fact that BoNT-A is a recommended first-line pharmacological treatment of spasticity; a lack of access to specialists performing BoNT-A injections; or ineffectiveness of a first BoNT-A injection leading to BoNT-A treatment discontinuation or delays in BoNT-A administration. Either way, further investigations on the reasons behind the limited use of BoNT-A for spasticity in France are warranted.

In line with our study results regarding the low adherence to BoNT-A therapy, a recent retrospective study from Düsseldorf, Germany performed among 1,351 patients treated with BoNT-A for different neurological indications found that more than half of patients discontinue BoNT-A within the first 8 years (32). The authors attribute this finding to a loss of interest due to the patients’ symptoms not being adequately relieved by BoNT-A therapy; successful accomplishment of therapy goals; patients’ inability to attend clinic appointments due to logistical problems (e.g., transportation); shorter therapy duration due to advanced age, earlier death, or worsening disability (e.g., from additional strokes or other comorbidities); or a combination of all of the above (32). To optimise adherence to BoNT-A therapy over time and to improve the overall outcome of rehabilitation following post-stroke spasticity, the use of adjunctive treatment including oral medication, physical therapy, occupational therapy, other neurolysis, and surgical intervention has been suggested (33, 34). Indeed, BoNT-A therapy in association with physical or occupational therapy and other multimodal approaches has been associated with improved patient outcomes and enhanced quality of life after CNS events like stroke (35, 36). To increase the use of BoNT-A in association with physical or occupational therapy and adjunctive treatments, continuous medical education programmes and patient information sessions can help disseminate knowledge about the advantages of this comprehensive treatment approach. In addition, establishing multidisciplinary care teams that include neurologists, rehabilitation specialists, physical therapists, occupational therapists, and orthotists can facilitate a collaborative approach to patient care and can ensure that BoNT-A injections are integrated with other rehabilitation therapies.

The median time between stroke onset and the first BoNT-A injection in the current analysis was 285 days or 9.4 months. This time interval seems to be long compared to other observational studies evaluating BoNT-A use for post-stroke spasticity, in which the average time between stroke onset and the first BoNT-A injection was 3–4 months (37, 38). The long interval between stroke onset and the first BoNT-A injection in our analysis may be due to several reasons, such as delays in accessing BoNT-A treatment resulting from logistical challenges, as well as the delayed diagnosis of post-stroke disabling spasticity. Indeed, it can take time for healthcare professionals to recognise and assess the extent of spasticity in stroke survivors, leading to a delay in initiating BoNT-A treatment (39). Moreover, in some cases of post-stroke spasticity, healthcare providers may adopt a conservative treatment approach, utilising other interventions like physical therapy, occupational therapy, or oral medications before resorting to BoNT-A injections. This stepwise approach could result in a delay between stroke onset and the first BoNT-A injection (34).

It is essential to emphasise that the appropriate timing of BoNT-A injections for spasticity treatment after a stroke depends on individual patient circumstances and on the professional judgment of the medical team involved in their care (17). However, as spasticity is a form of maladaptive plasticity that progresses over time, early initiation of BoNT-A therapy is generally encouraged to prevent or reduce neurological changes that lead to disabling spasticity (40, 41). Indeed, a longitudinal cohort study from Italy performed among 83 patients with post-stroke spasticity revealed that BoNT-A should be initiated within 3 months after stroke onset in order to obtain a greater reduction in muscle tone at 1 and 3 months afterwards (37). Nevertheless, the multinational, multicentre, observational early-BIRD study (42), which evaluated the real-world effectiveness of BoNT-A in patients with post-stroke upper-limb spasticity according to the time from stroke event to start of BoNT-A, found that BoNT-A treatment was consistently effective in reducing spasticity, whether started early (0–7 months) after the stroke event or later (36–443 months). These findings highlight the continued benefit of repeated BoNT-A injections at various disease stages (42). It is however likely that the early initiation of BoNT-A treatment may improve the outcomes of post-stroke spasticity. Importantly, BoNT-A treatment and follow-up should be offered as needed, without any limitations due to the duration of post-stroke spasticity (41). Routine evaluations for spasticity following a stroke should also be done indefinitely. Expert physicians agree that for patients treated with BoNT-A, a follow-up visit should be considered 4–6 weeks after each BoNT-A injection (41).

Our analysis also noted that the younger the patients with stroke, the more likely they were treated with BoNT-A injections. Older people are more likely to be affected by peripheral neuropathies, which may translate into a reduced spasticity prevalence in the elderly (43). Indeed, in a cross-sectional study from Sweden assessing the prevalence of disabling spasticity one year after first-ever stroke, younger age was associated with an increased risk of post-stroke spasticity (44). Younger patients with stroke are also more likely to receive BoNT-A injections than their older counterparts, as a consequence of the stroke care pathway adopted in geriatric rehabilitation, which in France differs from the care pathway adopted for younger stroke survivors. In geriatric rehabilitation in France, a more palliative approach is usually adopted, with fewer pharmacological interventions for stroke-related impairments such as spasticity.

In addition, our analysis identified a notably higher number of BoNT-A injections performed in acute care facilities compared to postoperative/rehabilitation facilities. However, in 2020, the proportion of BoNT-A injections performed in public postoperative/rehabilitation facilities substantially increased at the expense of public acute care outpatient care, for which the number of BoNT-A injections reached its peak in 2019. These observations may be related to recent postoperative/rehabilitation activity funding reforms in France including improved BoNT-A reimbursement mechanisms in postoperative/rehabilitation facilities, which are likely to lead to a further increase in the number of BoNT-A-treated patients as well as BoNT-A injections within postoperative/rehabilitation facilities compared to acute care facilities.

There were some limitations to the present study, inherent to retrospective electronic health record data analysis (based on ICD-10 and CCAM codes), including potential bias, missing data, and most importantly coding errors, as coding was made by the treating physicians and hence relies solely on physicians’ accuracy in selecting medical codes. Of note, current ICD-10 coding does not differentiate between focal and generalised spasticity, reducing accurate symptom tracking. Moreover, the quality of PMSI data depends on the accuracy and completeness of the information system. PMSI data usually relate to events such as hospital admissions or attendances and not individual patients. Another limitation of the PMSI database is that it does not contain data from outpatients discharged from private hospitals. Furthermore, we did not have access to data regarding BoNT-A dosage or effectiveness. Nevertheless, our analysis is strengthened by a large, representative, and contemporary patient population, with exhaustive data collection on BoNT-A use. To the best of our knowledge, this study is one of the few studies to have used a national exhaustive database to explore real-life BoNT-A treatment practices. Such approach minimises selection and reporting biases inherent to expert center cohorts and questionnaire-based studies, while contributing to improve clinical practice.

## 5. Conclusion

This population-based analysis of the exhaustive PMSI database highlights a broad range of BoNT-A treatment practices in France between 2014 and 2020. Although BoNT-A is a recommended first-line pharmacological treatment of spasticity that should be injected once every 3–4 months, BoNT-A treatment initiation and re-administration were low in the present study, particularly in patients with post-stroke spasticity. Further research into the limited use of BoNT-A among patients with CNS lesions in France is warranted. Future investigations should also target specific actions to improve spasticity-related care pathway.

## 6. Plain language summary

Botulinum toxin type A (BoNT-A) is a drug recommended to treat stiffness (spasticity) caused by various brain and spinal cord diseases, such as stroke. Usually, patients with spasticity require BoNT-A injections once every 3 to 4 months. Using electronic health records from a national hospital registry, we aimed to describe BoNT-A use in clinical practice in France between 2014 and 2020. Between 2014 and 2020, a total of 138,481 patients received at least one BoNT-A injection. However, during these years, 54% of these patients received only one or two BoNT-A injections. The number of patients who received BoNT-A to treat spasticity caused by brain and spinal cord diseases notably increased between 2014 and 2019 by an average of 8% per year, but between 2019 and 2020, this number remained stable probably due to the COVID-19 outbreak. In this analysis, spasticity affected 11% of stroke survivors. Between 2014 and 2020, only 2% of stroke survivors received at least one BoNT-A injection, and less than 1% of stroke survivors received BoNT-A injections once every 3 to 4 months. In summary, the implementation of BoNT-A treatment recommendations is not optimal in France. Spasticity management should thus be improved, with further research on the reasons for the limited use of BoNT-A for spasticity.

## Data availability statement

The data analyzed in this study was obtained from the Agence Technique de l'Information sur l'Hospitalisation (ATIH), the following licenses/restrictions apply: the datasets were provided under contract to Ipsen. Requests to access these datasets should be directed to JL, jonathan.levy2@aphp.fr.

## Ethics statement

Ethical approval was not required for the study involving humans in accordance with the local legislation and institutional requirements. Written informed consent to participate in this study was not required from the participants or the participants’ legal guardians/next of kin in accordance with the national legislation and the institutional requirements.

## Author contributions

JL, PK, AF, J-YL, and DB conceptualised and designed the study. PK acquired and analysed the data. All authors contributed to the article and approved the submitted version.

## Funding

This study was funded by Ipsen.

## Conflict of interest

JL is a consultant for Ipsen, Allergan, and Medtronic. PK is a consultant for Ipsen. AF and J-YL are Ipsen employees. DB is a consultant for Ipsen, Merz, Allergan, and Medtronic.

## Publisher’s note

All claims expressed in this article are solely those of the authors and do not necessarily represent those of their affiliated organizations, or those of the publisher, the editors and the reviewers. Any product that may be evaluated in this article, or claim that may be made by its manufacturer, is not guaranteed or endorsed by the publisher.
